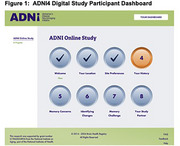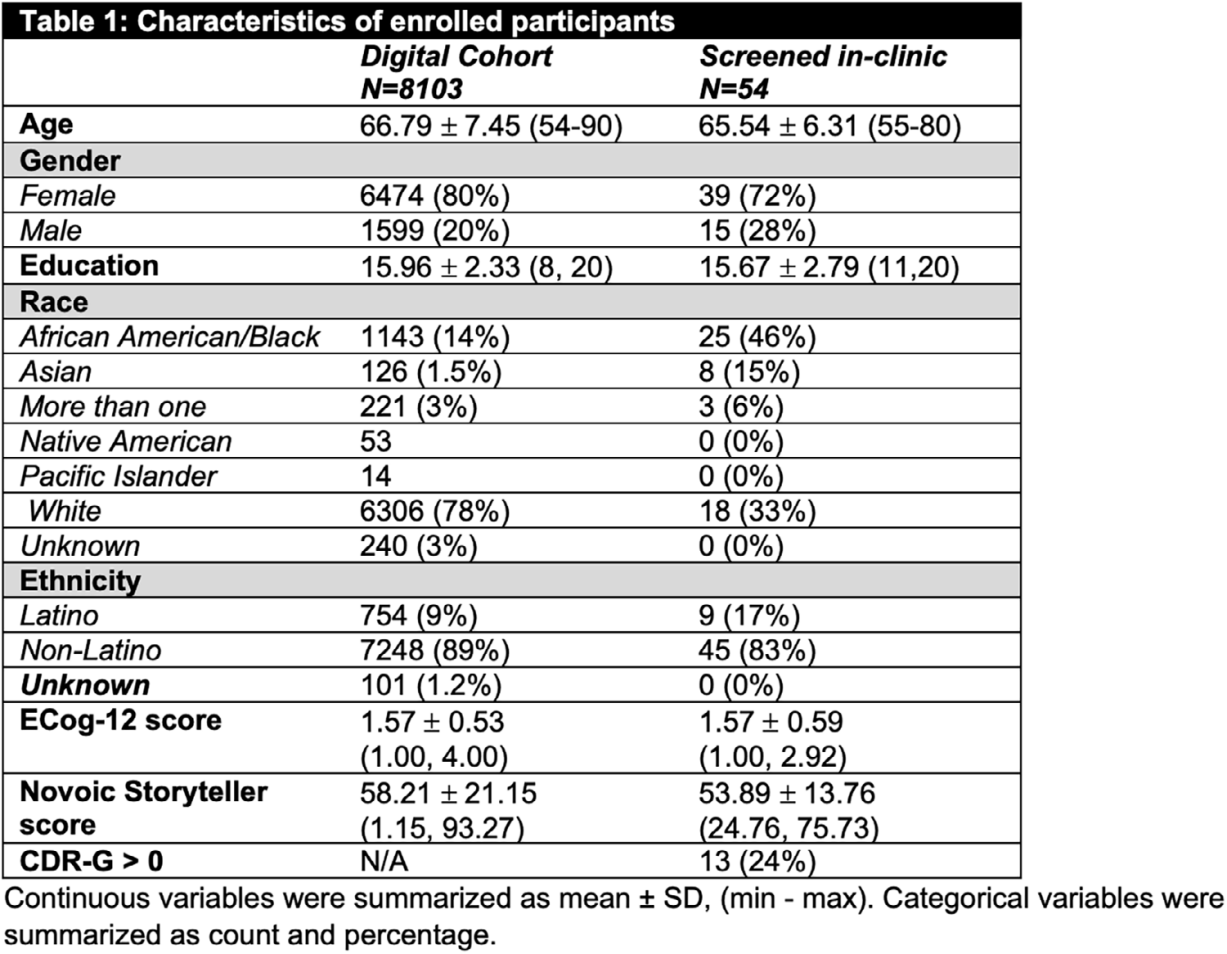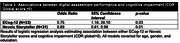# The ADNI4 Digital Study: Remote, unsupervised cognitive assessment to identify cognitive impairment in digitally recruited populations

**DOI:** 10.1002/alz70857_099537

**Published:** 2025-12-24

**Authors:** Melanie J. Miller, Naomi Saito, Catherine C. Conti, Derek Flenniken, Juliet Fockler, Diana Truran‐Sacrey, Adam Diaz, Jack Weston, Emil Fristed, Sarah Tomaszewski Farias, Danielle J. Harvey, Laurel Beckett, Ozioma C. Okonkwo, Monica G Rivera Mindt, Michael S. W. Weiner, Rachel L. Nosheny

**Affiliations:** ^1^ Northern California Institute for Research and Education (NCIRE), San Francisco, CA, USA; ^2^ University of California, Davis, Davis, CA, USA; ^3^ University of California, San Francisco, San Francisco, CA, USA; ^4^ Novoic, London, United Kingdom; ^5^ University of California, Davis School of Medicine, Sacramento, CA, USA; ^6^ University of Wisconsin, Madison, WI, USA; ^7^ Fordham University, New York, NY, USA

## Abstract

**Background:**

Scalable, efficient methods are needed to enroll older adults, especially those with Mild Cognitive Impairment (MCI), into AD observational studies and clinical trials. We evaluated feasibility of a novel, digital approach to recruit and screen participants for the Alzheimer's Disease Neuroimaging Initiative (ADNI4).

**Method:**

Digital advertising tailored towards older adults residing near clinical ADNI sites directed potential participants to a recruitment website to enroll in a Digital Cohort (Figure 1). They completed remote, unsupervised, digital surveys (demographics, ADNI exclusion criteria, memory concerns and changes, self‐report cognitive impairment, and the Everyday Cognition Scale (ECog)‐12 item); and the Novoic Storyteller self‐administered speech‐based cognitive test. Digital assessment results were used to prioritize those with possible MCI or dementia (based on self‐report cognitive impairment, ECog, and/or Novoic Storyteller scores), to ADNI sites for screening into in‐clinic ADNI. For those screened at ADNI sites, associations between ECog12 or Storyteller score and cognitive impairment assessed using the Clinical Dementia Rating global score were estimated using logistic regression.

**Result:**

Since June 2023, 8103 participants enrolled in the Digital Cohort (Table 1). Of those, 26% indicated ADNI4 exclusions (such as metal precluding MRI), 93% completed ECog12,16% had study partners who completed ECog12, and 60% completed Storyteller. For referral to clinical sites, 693 were invited, 313 accepted the invitation and were referred to a site, and 54 were screened at one of 44 ADNI sites (Table 1). In a subset with CDR screening data, more report of subjective cognitive decline on ECog12 and worse performance on Storyteller were associated with greater odds of cognitive impairment (CDR global >0) (Table 2).

**Conclusion:**

Recruitment and assessment of older adults, including those with possible cognitive impairment, is feasible using tailored digital advertising and remote, unsupervised digital assessment. Remote assessments can be used to enrich for cognitive impairment. This approach can be adapted to facilitate recruitment and longitudinal assessment in other AD studies and trials